# Arabidopsis Calcium Dependent Protein Kinase 3, and Its Orthologues OsCPK1, OsCPK15, and AcCPK16, Are Involved in Biotic and Abiotic Stresses

**DOI:** 10.3390/plants14020294

**Published:** 2025-01-20

**Authors:** Gardette R. Valmonte-Cortes, Colleen M. Higgins, Robin M. MacDiarmid

**Affiliations:** 1 School of Science, AUT City Campus, Auckland University of Technology, Auckland 1142, New Zealand; colleen.higgins@aut.ac.nz; 2 The New Zealand Institute for Plant & Food Research Limited, 120 Mt Albert Road, Auckland 1025, New Zealand; robin.macdiarmid@plantandfood.co.nz; 3 School of Biological Sciences, The University of Auckland, Thomas Building, 3a Symonds Street, Auckland 1010, New Zealand

**Keywords:** calcium-dependent protein kinase, *AtCPK3*, *OsCPK1*, drought response, pathogen response

## Abstract

Calcium-dependent protein kinases (CPKs) are plant proteins that directly bind calcium ions before phosphorylating substrates involved in biotic and abiotic stress responses, as well as development. *Arabidopsis thaliana* CPK3 (*AtCPK3*) is involved with plant signaling pathways such as stomatal movement regulation, salt stress response, apoptosis, seed germination and pathogen defense. In this study, *AtCPK3* and its orthologues in relatively distant plant species such as rice (*Oryza sativa*, monocot) and kiwifruit (*Actinidia chinensis*, asterid eudicot) were analyzed in response to drought, bacteria, fungi, and virus infections. Two orthologues were studied in *O. sativa*, namely *OsCPK1* and *OsCPK15*, while one orthologue—*AcCPK16*—was identified in *A. chinensis*. Reverse-transcriptase quantitative PCR (RT-qPCR) analysis revealed that *OsCPK1* and *AcCPK16* exhibit similar responses to stressors to *AtCPK3*. *OsCPK15* responded differently, particularly in bacterial and fungal infections. An increase in expression was consistently observed among *AtCPK3* and its orthologues in response to virus infection. Overexpression mutants in both Arabidopsis and kiwifruit showed slight tolerance to drought, while knockout mutants were slightly more susceptible or had little difference with wild-type plants. Overexpression mutants in Arabidopsis showed slight tolerance to virus infection. These findings highlight the importance of *AtCPK3* and its orthologues in drought and pathogen responses and suggest such function must be conserved in its orthologues in a wide range of plants.

## 1. Introduction

How plants respond to stress is of great importance due to global challenges in crop productivity and environmental sustainability [1,2,3,4]. These stressors include both abiotic factors (such as drought, extreme temperatures, high salinity, and nutrient starvation) and biotic factors (including bacterial, viral, and fungal infections, as well as insect herbivores and nematode pests). Plants have developed signaling networks and regulatory pathways that help them perceive and withstand stress and infections [5,6,7]. One important component of plant stress response is calcium (Ca^2+^) signaling, which also plays a fundamental role in plant growth and development [8,9,10].

Cytosolic Ca^2+^ levels elevate in complex spatio-temporal patterns called ‘Ca^2+^ signatures’ in response to various developmental and stress stimuli [11,12]. These signatures are detected by a vast array of Ca^2+^-sensors and responder proteins, such as calmodulins (CaMs), calmodulin-like proteins (CaMLs), Ca^2+^/CAM-dependent protein kinases (CaMKs), Ca^2+^ and Ca^2+^/CAM-dependent protein kinases (CCaMKs), Calcineurin B-like proteins (CBLs), CBL-interacting protein kinases (CIPKs), and calcium-dependent protein kinases (CPKs) [12,13]. These proteins undergo conformational changes upon binding with Ca^2+^ and consequently transmit specific signals to their substrates through phosphorylation [12,13].

Among proteins involved in calcium signaling, CPKs are unique because they have both a Ca^2+^ sensor domain that directly binds Ca^2+^ ions and a responder (protein kinase) domain that phosphorylates specific protein targets [14]. CPKs are involved in diverse functions, including growth, development and stress response. Genome-wide studies in different plant and algal species identified variable sizes of the CPK gene family, ranging from two to about a hundred [15,16,17,18,19,20,21,22,23,24,25,26,27,28,29,30,31,32,33,34,35,36,37,38,39]. CPKs are mostly expressed throughout the plant, while some are only expressed in specific tissue types. CPK genes are highly conserved, which correlates with their functional overlapping and redundancy [15,38,40]. Subcellular localization of CPKs also varies and is often associated with cellular membranes such as the plasma membrane, endoplasmic reticulum, and outer mitochondrial membrane, as well as other structures in the cytoplasm and the nucleus [41,42,43]. Many CPKs appear to play important roles in cellular signaling pathways that bring about tolerance to drought, salt, heat and cold, as well as defense pathways against pathogens and herbivores [18,24,39,42,44,45,46,47,48,49,50,51,52,53,54].

Arabidopsis (*Arabidopsis thaliana*) CPK3 (At4g23650) appears to have key roles in various biotic and abiotic stress responses, as well as development. It is involved with methyl jasmonate signaling and ABA regulation of guard cell channels and stomatal closure [51,55,56,57,58] and promotes salt stress acclimation [59], which is vital for plant survival in drought and abiotic stress conditions. Furthermore, *AtCPK3* is vital to plant immune responses as it is required for sphingolipid-induced cell death [60], increases promoter activity of a flg-22 responsive gene [45], promotes plant defensin gene transcription during a herbivore attack [47], and regulates actin cytoskeleton organization and immunity [61].

While the important roles of *AtCPK3* have been well studied in Arabidopsis, little is known if its function is maintained in its orthologues in other plant species. This report presents the responses of *AtCPK3* and its orthologues in important crops that also represent other major plant groups: rice (*Oryza sativa*), a monocot species and kiwifruit (*Actinidia chinensis*), a eudicot species belonging to the Asterid clade, which is different from Arabidopsis (Rosid).

## 2. Results

### 2.1. OsCPK1, OsCPK15 and AcCPK16 Identified as Orthologues of Arabidopsis AtCPK3 in Rice and Kiwifruit

**Figure 1:**
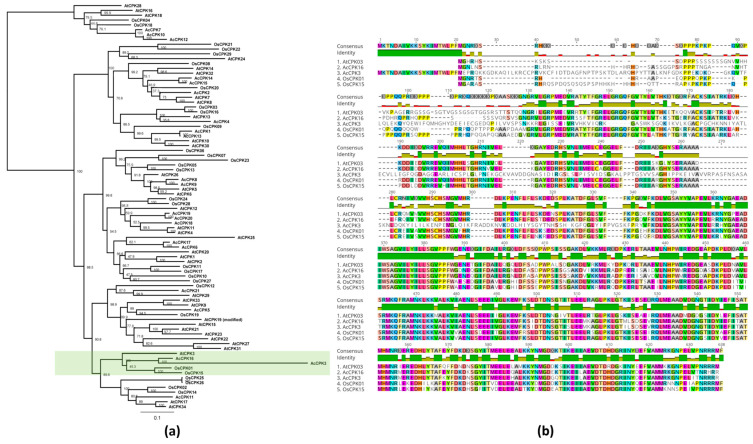
Phylogenetic analysis and sequence alignment of Arabidopsis (*Arabidopsis thaliana*) CPK3 (*AtCPK3)* and its orthologues in rice (*Oryza sativa*) and kiwifruit (*Actinidia chinensis*). (**a**) Phylogenetic analysis of Arabidopsis, rice, and kiwifruit CPKs. Group IIb CPKs are highlighted in green. (**b**) Multiple alignments of Group IIb CPK amino acid sequence. Positions with amino acid similarities are highlighted with the same color.

A Maximum Likelihood analysis of all known CPK protein sequences from *A. thaliana*, *O. sativa,* and *A. chinensis* was used to determine the orthologues of *AtCPK3* in the two plant species (Figure 1a). These were identified as *OsCPK01* (Os01g43410) and *OsCPK15* (Os05g50810) in rice and *AcCPK16* (DB Acc No. 5527801) in kiwifruit. The orthologues of *AtCPK3* in rice can also be identified from previous studies of CPKs from *O. sativa* [16,62]. The apparent protein orthologues of AtCPK3 in kiwifruit were AcCPK16 and AcCPK3. AcCPK16 has 69.89% aa identity with AtCPK3, while AcCPK3 only has 45.39% aa identity, as its 5′ half did not align well with either AcCPK16 or AtCPK3. This could be due to issues with the splicing algorithms utilized in gene prediction software. Hence, the latter analysis focused on AcCPK16 and did not include AcCPK3.

### 2.2. Transcript Accumulation of AtCPK3 and Its Orthologues in Rice and Kiwifruit Change in Response to Abiotic and Biotic Stressors

#### 2.2.1. Drought

**Figure 2:**
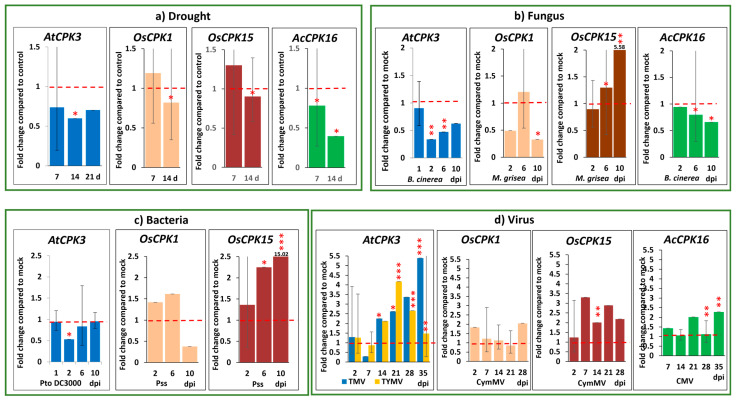
Transcriptional expression levels of *AtCPK3* and its orthologues *OsCPK1*, *OsCPK15* and *AcCPK16* in response to biotic and abiotic stress by RT-qPCR. (**a**) Drought (**b**) fungus (**c**) bacteria (**d**) virus. d, days; dpi, days post inoculation; Pto DC3000, *Pseudomonas syringae* pv*. tomato* DC3000*;* Pss, *P. syringae* pv*. syringae;* TMV, tobacco mosaic virus; TYMV, turnip yellow mosaic virus; CymMV, cymbidium mosaic virus; CMV, cucumber mosaic virus. Red broken lines indicate expression level in negative control or mock-inoculated plants, normalized at 1. Error bars indicate standard error values after normalization, log transformation and mean centering, using *AtSAND*, *OsEP1*, and *AcACTIN* as reference genes, respectively. Statistical support is indicated as strong (***, *p* ≤ 0.01), good (**, 0.01 < *p* < ~0.05) or weak (*, 0.05 < *p* < ~0.10) as per ANOVA test followed by Fisher’s LSD and/or Tukey test.

*AtCPK3* and its orthologues in rice and kiwifruit generally showed a decrease in transcript accumulation in response to drought at 14 days, with some fluctuations at 7 days (Figure 2a). In Arabidopsis leaves, *AtCPK3* exhibited a significant decrease in transcript, nearly halving under drought conditions at 14 and 21 days compared to controls, although the statistical support was weak (Figure 2a). In rice, both *OsCPK1* and *OsCPK15* slightly decreased at 14 days. In kiwifruit, *AcCPK16* decreased at both 7 and 14 days.

#### 2.2.2. Fungus

*AtCPK3*, *OsCPK1*, and *AcCPK16* transcript accumulation also decreased following infection with fungal pathogens *Botrytis cinerea* or *Magnaporthe grisea*, but not *OsCPK15* (Figure 2b). *AtCPK3* decreased by about 1.5-fold in Arabidopsis response to *B. cinerea* at 2 and 6 dpi. In a detached leaf infection of rice with *M. grisea, OsCPK1* decreased 1.3-fold at 10 dpi. On the other hand, *OsCPK15* increased by about two-fold at 6 and 10 dpi. In kiwifruit, *AcCPK16* exhibited only a slight decrease at day 10 in response to *B. cinerea*.

#### 2.2.3. Bacteria

*AtCPK3* also showed a slight decrease in transcript accumulation in response to infection with the bacterial pathogen *Pseudomonas syringae* (Pto DC3000), but results were not consistent for *OsCPK1* and *OsCPK15* (Figure 2c). There was a decrease in *AtCPK3* transcript at 2 dpi in response to *P. syringae* infection. *OsCPK1* showed a slight decrease at 10 dpi, but this was not statistically significant. *OsCPK15*, on the other hand, increased by about two-fold at 6 dpi and about 15-fold at 10 dpi. Bacterial infection in kiwifruit was not performed due to restrictions in biological security.

#### 2.2.4. Virus

*AtCPK3* and its orthologues in rice and kiwifruit showed an increase in transcript accumulation in response to different viruses across a series of time points (Figure 2d). In response to tobacco mosaic virus (TMV) and turnip yellow mosaic virus (TYMV), *AtCPK3* increased at 14, 21, 28, and 35 days post inoculation (dpi). Cymbidium mosaic virus (CymMV), a common virus affecting orchids and other plants in the Poaceae family [63,64], was used to infect rice plants. *OsCPK1* and *OsCPK15* showed increased transcript accumulation in response to CymMV. Cucumber mosaic virus (CMV), which infects *Actinidia* species [65], was used for kiwifruit. Similarly, *AcCPK16* showed increased transcript accumulation in response to CMV at 28 and 35 dpi of CMV infection.

### 2.3. Overexpression of AtCPK3 and AcCPK16 Confers Some Tolerance to Drought

**Figure 3:**
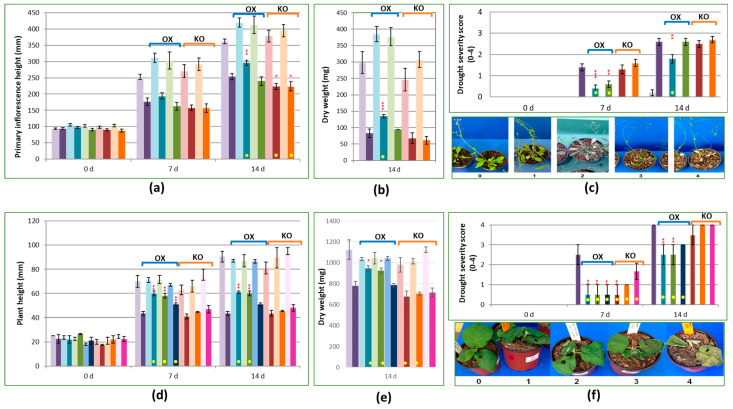
Phenotype analysis of the wild-type and transgenic lines of Arabidopsis (*Arabidopsis thaliana*) and kiwifruit (*Actinidia chinensis*) under drought stress (non-watering). (**a**) Primary inflorescence height of Arabidopsis lines at 0, 7 and 14 dpi. (**b**) Dry weights of Arabidopsis lines; samples dried after 14 days of non-watering. (**c**) The drought severity score among Arabidopsis lines is shown with a scoring reference at the bottom. (**d**) Height of kiwifruit lines at 0, 7 and 14 dpi. (**e**) Dry weights of kiwifruit lines; samples dried after 14 days of non-watering. (**f**) The drought severity score among kiwifruit lines is shown with a scoring reference at the bottom. OX, overexpression mutant lines; KO, knockout mutant lines. The color of the bars matches the plant lines. For (**a**–**c**): purple, Wild-type Arabidopsis (*col-0*); blue, *AtCPK3* OX (SAIL-120-H09); green, *AtCPK3* OX (pHex2AtCPK3.3); red, *atcpk3-1* KO (SALK_106720C); and orange, *atcpk3-2* KO (SALK_022862); For (**d**–**f**): purple, Wild-type kiwifruit; blue, *AcCPK16* OX E05; green, *AcCPK16* OX E06; dark blue, *AcCPK16* OX E07; red, *AcCPK16* KO E05; orange, *AcCPK16* KO E10; and pink, *AcCPK16* KO E11. Bars with lighter colors indicate control plants, while bars with darker colors indicate plants subjected to non-watering. Yellow dots indicate a marked difference between the transgenic line and the wild-type plants. Statistical support is indicated as strong (***, *p* ≤ 0.01), good (**, 0.01 < *p* < ~0.05) or weak (*, 0.05 < *p* < ~0.10) as per ANOVA test followed by Fisher’s LSD and/or Tukey test.

Wild-type Arabidopsis, two *AtCPK3* OX lines (SAIL_120_H09 and pHEX2AtCPK3) and two *AtCPK3* KO lines (*atcpk3-1* and *atcpk3-2*) were subjected to drought treatment for 14 days. Plant height (primary inflorescence), dry weights and severity scores of 10 plants per treatment for each line were measured to compare their phenotypic responses to drought conditions (Figure 3a, b, and c, respectively). Significant reductions in plant height and dry weights at 7 and 14 d were observed in plants subjected to drought treatment compared to those under control conditions.

At 14 days of drought treatment, the overexpressor line SAIL_120_H09 appeared to be marginally taller than WT, whereas *atcpk3-1* and *atcpk3-2* plants appeared to be marginally shorter than wild-type plants. pHEX2AtCPK3 overexpressor plants did not show a significant difference in height compared to WT. With regards to dry weights, the overexpressor lines showed higher values, though only the SAIL_120_H09 line showed a significant difference. The knockout plants *atcpk3-1* and *atcpk3-2* showed slightly lower dry weights than WT. Severity scores at 7 d among SAIL_120_H09 and pHEX2AtCPK3 plants were markedly less than WT, while *atcpk3-1* and *atcpk3-2* plants did not differ from WT (Figure 3c). At 14 d, only SAIL_120_H09 showed significantly lower severity scores than WT.

As there are significant overlaps between drought and salt tolerance, salt tolerance was also studied (Supplementary Figures S1 and S2). Seed germination at 150 mM salt treatment was significantly higher among all transgenic lines compared to the wild-type seeds. Wild-type seeds showed only 20% seed germination, while both overexpressor lines showed about 60% seed germination rate. Interestingly, knockout lines demonstrated mild tolerance, with about 30% seed germination.

Wild-type kiwifruit, three *AcCPK16* OX lines (*AcCPK16* OX 05, 06 and 07) and three *AcCPK16* KO lines (*AcCPK16* KO 05, 10 and 117) were subjected to drought treatment for 14 days. Similar to Arabidopsis, marked reductions in plant height and dry weights at 7 and 14 dpi were observed in kiwifruit plants subjected to drought treatment compared to those under control conditions (Figure 3d,e). At 7 and 14 d, all three *AcCPK16* OX lines were marginally taller than WT, while the *AcCPK16* KO lines did not show a significant difference from WT.

Interestingly, there was very little difference in the dry weights of the control and drought treatment groups among *AcCPK16* OX and KO lines. *AcCPK16* OX E05 and E06 also showed marginally higher dry weights than WT, while *AcCPK16* KO E10 plants showed marginally lower dry weights than WT.

Severity scores for drought at 7 d were lower than WT among all *AcCPK16* OX lines and two KO lines, E05 and E10 (Figure 3f). All these five lines showed good statistical support. At 14 d, the severity scores of *AcCPK16* OX lines were significantly lower than WT, whereas *AcCPK16* KO lines did not show a significant difference.

### 2.4. Overexpression of AtCPK3 Potentially Confers Some Tolerance to Virus Infection

**Figure 4:**
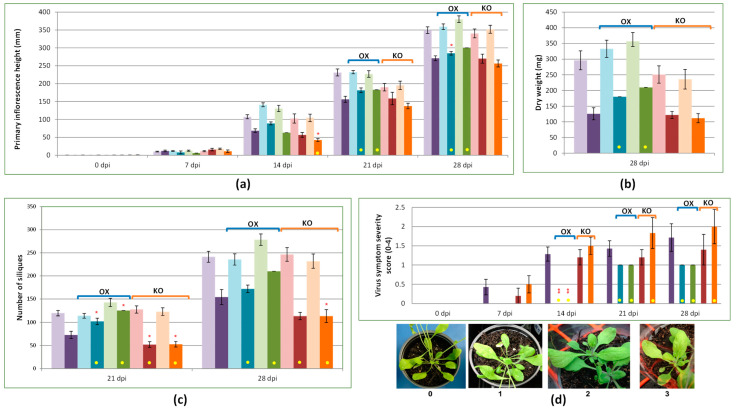
Phenotype analysis of the wild-type and transgenic lines of Arabidopsis (*Arabidopsis thaliana*) virus infection (TYMV). (**a**) Primary inflorescence height of Arabidopsis lines at 0, 7, 14, 21 and 28 dpi. (**b**) Dry weights of Arabidopsis lines; samples dried after 28 days of inoculation. (**c**) Number of siliques of Arabidopsis lines at 21 and 28 dpi. (**d**) Virus symptom severity, with scoring reference below. OX, overexpression mutant lines; KO, knockout mutant lines. Color of bars match the plant lines: purple, Wild-type Arabidopsis (*col-0*); blue, *AtCPK3* OX (SAIL-120-H09); green, *AtCPK3* OX (pHex2AtCPK3.3); red, *atcpk3-1* KO (SALK_106720C); and orange, *atcpk3-2* KO (SALK_022862). Bars with lighter colors indicate mock-inoculated plants, while bars with darker colors indicate plants infected with TYMV. Yellow dots indicate a marked difference between the transgenic line and the wild-type plants. Statistical support is indicated as strong, good (**, 0.01 < *p* < ~0.05) or weak (*, 0.05 < *p* < ~0.10) as per ANOVA test followed by Fisher’s LSD and/or Tukey test.

Wild-type Arabidopsis, two *AtCPK3* OX lines (SAIL_120_H09 and pHEX2AtCPK3.3), and two *AtCPK3* KO lines (*atcpk3-1* and *atcpk3-2*) were compared in terms of their phenotypic responses to TYMV (Figure 4). Only a few plants were successfully infected (out of 10 plants per group): seven wild-type plants, two SAIL-120_H09 plants, one pHEX2AtCPK3 plant, five *atcpk3-1* plants and six *atcpk3-2* plants. Only mild symptoms were observed generally. Plant height (primary inflorescence), severity scores, and dry weights of these plants were compared.

Marked reductions in plant height at 14, 21, and 28 dpi were mostly observed in plants infected with TYMV compared to the mock-inoculated plants (Figure 4a). At 14 dpi, SAIL_120_H09 plants (89.00 mm) were taller than WT (68.71 mm), whereas *atcpk3*-2 plants (42.5 mm) were shorter than WT. At 21 and 28 dpi, both SAIL_120_H09 (181.5 mm and 285 mm) and pHEX2AtCPK3 (183.00 mm and 300.00) plants were marginally taller than WT (156.29 mm and 271.00 mm), while the *atcpk3-2* plants (137.67 mm and 256.50 mm) were marginally shorter than WT. Only the marked difference between SAIL_120_H09 and WT at 28 dpi showed good statistical support.

SAIL_120_H09 and pHEX2AtCPK3 showed heavier dry weights at 28 dpi (180.0 and 210.0 mg) than WT (125.7 mg), while *atcpk3-1* and *atcpk3-2* plants did not show a marked difference (122.0 and 111.7 mg) from WT (Figure 4b). However, the marked differences in dry weights were not statistically supported.

The overexpressors SAIL_120_H09 and pHEX2AtCPK3 produced more siliques than WT at 21 and 28 dpi, while the knockout plants *atcpk3-1* and *atcpk3-2* had fewer siliques (Figure 4c).

Virus infection symptoms only started becoming observable in SAIL_120_H09 and pHEX2AtCPK3 at 21 and 28 dpi and were less severe than WT (Figure 4d). The knockout lines *atcpk3-1* and *atcpk3-2* did not show a marked difference in severity from WT, although *atcpk3-2* appeared to be slightly more severe. Good statistical evidence to support the marked difference from WT was only observed among SAIL_120_H09 and pHEX2AtCPK3 lines at 14 dpi.

### 2.5. Overexpression of AtCPK3 and AcCPK16 in Response to Fungal Infection

Symptom scores were gathered to measure the response of Arabidopsis plants to *B. cinerea* (Supplementary Figure S3a). No marked difference was observed between the wild-type and all the transgenic plants, except for SAIL_120_H09 overexpressors, which had marginally lower mean scores at 7 dpi (with weak statistical support).

Detached leaf cuttings of wild-type kiwifruit, *AcCPK16* OX 05, and three *AcCPK16* knockout lines (*AcCPK16* KO 05, 10 and 11) were spot inoculated with *B. cinerea*. The area of lesions with fungal growth on the leaves was measured at 2 and 7 dpi (Supplementary Figure S3b). At 2 dpi, no marked differences in fungal growth area among any lines were observed, except for KO E05, which had marginally larger lesions than WT. At 7 dpi, *AcCPK16* OX E05 leaves showed markedly greater fungal growth compared to all WT and KO leaves. This was supported by good statistical evidence. No significant difference in fungal growth area was observed among WT and KO leaves.

## 3. Discussion

This study highlights the potential functional orthologues of *AtCPK3* in the crop plants rice and kiwifruit. *AtCPK3* belongs to Group IIb CPKs, considered to be the most conserved group of CPKs based on a phylogenetic analysis using amino acid sequences of CPKs from various plants [38]. Given the high likelihood of functional similarity among highly conserved sequences and considering *AtCPK3*’s known role in various biotic and abiotic responses, identifying and characterizing its orthologues in other plants can offer valuable insights into CPK function and evolution. Additionally, these orthologues can serve as promising gene targets for agricultural applications. Our findings suggest that *AtCPK3* orthologues in a monocot species (*O. sativa*, *OsCPK1*) and an asterid (*A. chinensis, AcCPK16*) generally exhibit functional similarities.

Based on RT-qPCR experiments, *AtCPK3* and its orthologues in rice and kiwifruit exhibited downregulation in response to drought, bacteria and fungal infection, and upregulation in response to virus infection. Downregulation by about 0.5-fold under drought and fungal infections was observed, except for *OsCPK1*5, which was upregulated in response to fungal infection. A similar downregulation was observed with bacterial infections, except for *AcCPK16*, which was not tested due to containment restrictions. In contrast, all genes showed upregulation in response to viral infections. The statistical support for the RT-qPCR results was not always strong, likely due to the limited number of manageable samples for time-series experiments (n = 3 plants for each timepoint). However, the consistent trends observed across the tested stressors suggest a shared function between *AtCPK3*, *OsCPK1*, and *AcCPK16*.

Our experiments in transgenic Arabidopsis and kiwifruit plants revealed similar phenotypic responses to drought, supporting the involvement of *AtCPK3* in drought tolerance and the potential functional similarity of *AcCPK16* with *AtCPK3*. Both *AtCPK3* and *AcCPK16* overexpressor lines showed greater plant height, greater dry weight, and lower severity scores under drought conditions compared to wild-type plants. These findings are consistent with previous studies demonstrating the role of *AtCPK3* in drought tolerance through various mechanisms, including the regulation of stomatal closure by phosphorylating certain receptors [55,57,66,67]. *AtCPK3*, as well as *AtCPK6*, have been reported to be involved in ABA-regulated signaling that causes stomatal closure and reduced water loss under drought conditions, as *cpk3cpk6* double mutants showed partial impairment of stomatal closure [55]. Moreover, ABA and calcium activation of slow (S)-type anion channels (SLAC) was impaired in single and double *cpk3cpk6* mutant guard cells, which suggested that *AtCPK3* and *AtCPK6* are part of a phosphorylation cascade that opens S-type channels, allowing ions (and subsequently water) to move out of the guard cells and reduce its turgor pressure [55]. A subsequent study has investigated this further and found that *AtCPK3* regulates the interaction between the stomatal closing mediator, inositol-1,4,5-triphosphate (IP3) and S-type channels [57]. More recently, it was found that *AtCPK3* and *AtCPK6* phosphorylate stomatal closure-related actin-binding protein 1 (*SCAB1*), which is considered a molecular switch that destabilizes F-actin, leading to stomatal closure [67]. The enhanced drought tolerance observed in the overexpressor lines may be attributed to these mechanisms. Additionally, it has been suggested that *AtCPK3* and *AtCPK16* phosphorylate and stabilize the transcription factor *AtABF3* (Abscisic acid-responsive element binding factor 3), allowing it to activate the *ABI5* (ABA-Insensitive 5) gene in response to salt stress [68]. Aside from salt stress, *ABI5* is also involved in drought by activating the expression of various stress-responsive genes, integrating phytohormone signaling, and promoting stomatal closure [69,70]. It would be valuable to investigate these mechanisms in kiwifruit as the observed similarity in the phenotypic responses of *AtCPK3* and *AcCPK16* overexpressors suggests that they may share conserved mechanisms of action in drought conditions. Similarly, a functional analysis of soybean *GmCDPK3*, also an orthologue of *AtCPK3*, demonstrated that overexpression in Arabidopsis conferred tolerance to drought in terms of higher seed germination rates under polyethylene glycol (PEG6000) treatment [71]. Our findings regarding the seed germination rates of overexpressor lines under salt treatment are consistent with these.

Our knockout lines did not show significant differences from wild-type plants, suggesting functional redundancy among CPK family members. This redundancy is a well-documented phenomenon in plant signaling pathways, where multiple genes can compensate for the loss of function of a single gene, as was demonstrated with *AtCPK3* and *AtCPK6* in other studies mentioned above. Therefore, future experiments should consider generating multiple gene knockout lines to fully elucidate the roles of *AtCPK3* and its orthologues in drought tolerance. Furthermore, homozygous transgenic lines (both overexpressors and knockouts) at T4 generation or later will provide a better comparison. There may be challenges with the timeframe, as some plant species require a longer generation time, such as kiwifruit, which requires three to five years to produce fruit.

The enhanced drought tolerance among overexpressors may appear in contrast to the observed decrease in *AtCPK3* transcript levels in wild-type plants under drought conditions. It may be that the initial increase of *AtCPK3* transcript occurs earlier than 7 days, with sufficient protein levels maintained to confer drought tolerance, or that the reduction in *AtCPK3* transcript occurs as an impact of prolonged drought conditions. It is possible that while CPK3 is initially activated to mediate the rapid closure of stomata during drought, its activity is subsequently reduced to allow for a more balanced response to prolonged water deficit. Hence, future investigations looking at drought responses in a shorter timeframe will be useful.

The transcript accumulation and phenotype studies in response to bacteria, fungi, and viruses may correlate with the reported roles of *AtCPK3* in response to biotic stress. Arabidopsis CPK3 plays a critical role in sphingolipid long-chain base-mediated cell death, which is a vital process in plant defense responses by phosphorylating 14-3-3 proteins [60]. Additionally, it was demonstrated that *AtCPK3* phosphorylates *ADF4* (Actin-depolymerizing factor 4), which regulates actin cytoskeletal organization in stomatal guard cells and controls stomatal movement during pattern-triggered immunity (PTI) responses [61]. In *cpk3-2* mutants, the immune response is impaired, evidenced by increased pathogen growth (*P. syringae* DC3000 expressing AvrPphB) and the absence of the hypersensitive response (HR), indicating that *AtCPK3* is required for resistance to the pathogen [61]. It would be valuable to determine if this function is also exhibited by *AcCPK16*, such as in response to *P. syringae* pv. *actinidiae* (Psa) infections for which the stomata serve as one of the primary entry points. *AcCPK16* may play a crucial role in the kiwifruit plant’s defense mechanism by regulating stomatal closure to prevent pathogen entry, in addition to its potential to enhance drought tolerance.

The increase in transcript accumulation and the observed tolerance among *AtCPK3* overexpressors in response to the viruses tested supports *AtCPK3*’s important role in plant virus response. This is consistent with recent studies that explored the function of *AtCPK3* in phosphorylating remorin (REM) proteins, which resulted in reduced viral cell-to-cell movement in *Nicotiana benthamiana* [72] and *A. thaliana* [73]. REM proteins have been associated with biotic and abiotic stresses by regulating plasma membrane nanodomains, which are small, specialized regions (typically between 10 and 200 nanometers) surrounding the membrane that are enriched with specific lipids, proteins, and other molecules [74]. REM proteins are essential regulators of plant-microbe interactions [75] and have been reported to restrict Potato virus X (PVX) movement in tomatoes [76] and *N. benthamiana* [77]. Plant PVX sensing activates AtCPK3 protein, which phosphorylates group 1 REMs [72]. Transient and constitutive overexpression of *AtCPK3* in *N. benthamiana* resulted in reduced PVX spreading, while underexpression of group I REMs resulted in a reduction in the ability of *AtCPK3* overexpressors to restrict PVX movement [72]. In Arabidopsis infected with Plantago asiatica mosaic virus (PlAMV), *cpk3-1* and *cpk3-2* knockout lines showed enhanced local virus propagation (40–60%), while *AtCPK3* overexpressor lines showed some restriction in local propagation (10–20%), indicating the role of *AtCPK3* in restricting PIAMV propagation [73]. The association of AtCPK3 proteins with the plasma membrane is crucial to this function [73]. It would be worthwhile carrying out such an investigation of the orthologues of *AtCPK3* in rice (i.e., *OsCPK1*) and kiwifruit (i.e., *AcCPK16*) and the viruses that commonly threaten the production of these crops.

In response to *B. cinerea*, *AtCPK3* and *AcCPK16* OX lines appeared to have opposite responses. These findings were inconclusive, as the *AtCPK3* OX lines showed only a marginal degree of tolerance. Moreover, the *AcCPK16* OX lines in kiwifruit showed greater fungal growth at 7 dpi; this may be due to a higher amount of moisture in the detached leaves, promoting fungal growth. This may correlate with the drought tolerance observed among overexpressors but requires further investigation.

*OsCPK15*, while being highly similar in sequence to *OsCPK1*, seems to be a paralogous gene that has undergone neofunctionalization, as it exhibited opposite responses to the stressors, particularly transcript responses to bacterial and fungal infections. Amino acid variations between *OsCPK1* and *OsCPK15* were mostly found in the N-terminus, which is essential for cellular localization and specific function. This may explain the difference in their function, as variability in the N-terminal domain among different CPKs is known to contribute to their specific roles in cellular signaling and stress responses [48]. A few single amino acid differences were also found across the kinase domain, but none of those were located in the active sites. A comprehensive analysis of these two genes and their function is imperative. Construction and analysis of *OsCPK1* and *OsCPK15* single and double mutant lines (not included in this study due to limitations in containment permissions, genetic stock availability, and timeframe), as well as targeted mutations, will be valuable to further elucidate this. Transgenic rice overexpressing *OsCPK1* and *OsCPK15* (single and double mutants) will also be useful to further affirm drought and virus tolerance among overexpressors of *AtCPK3* orthologues.

Plant responses to biotic and abiotic stresses are interconnected through different molecular signaling pathways, such as calcium signaling, mitogen-activated protein kinase (MAPK) cascades, reactive oxygen species (ROS), and phytohormones like abscisic acid (ABA), salicylic acid (SA), jasmonic acid (JA), and ethylene (ET) [78]. The crosstalk between signaling pathways allows plants to efficiently manage multiple stresses simultaneously, enhancing their overall resilience [79,80,81]. Calcium sensors such as CPKs are considered key hubs linking biotic and abiotic stress responses [48,82]. *AtCPK3* serves as one such pivotal hub, particularly with its key role in stomatal closure and in interacting with other signaling molecules such as ROS and phytohormones. The dual role of *AtCPK3* in managing both pathogen invasion and drought stress highlights the interconnected nature of these stress responses and underscores the importance of stomatal regulation in plant immunity and resilience. Much of the link between *AtCPK3* and hormone signaling has been reported with regard to ABA responses. However, it is also possible that a connection with SA and JA signaling pathways may be determined by identifying defense-related genes and/or proteins that *AtCPK3* and its orthologues may influence.

These findings are highly valuable for advancing research into *AtCPK3* and its orthologues in crop plants. Our findings and other studies suggest that overexpressing these genes could enhance tolerance or resistance to plant diseases and environmental stress, particularly drought and virus infections. Alternatively, plants that naturally express higher levels of *AtCPK3* may exhibit greater tolerance and resistance to such challenges. As there is a shared function between *AtCPK3* and its orthologues in crop plants that are distantly related to Arabidopsis, this may also be conserved in a wide range of plant species.

## 4. Materials and Methods

### 4.1. Identifying AtCPK3, 17 and 34 Orthologues in Rice and Kiwifruit

The orthologues of *AtCPK3* in rice and kiwifruit were identified using phylogenetic analyses. For rice, CPK sequences were obtained from the phylogenetic trees reported by previous authors [16,62]. Since there was no previous report identifying CPKs from the kiwifruit genome [83], a BLAST search was carried out using all 34 AtCPK coding sequences as query sequences against three sets of databases available from The New Zealand Institute for Plant and Food Research Limited (PFR) Genome Server: (1) *A. chinensis* EST library; (2) *A. chinensis* CK15_02 Genome Scaffolds; and (3) *A. chinensis* CK51F3_01 Hybrid Gene Models. Kiwifruit sequences were named AcCPK1 to AcCPK21. Nucleotide and protein sequences of the AtCPKs, OsCPKs, and AcCPKs were aligned using the ClustalW program [84] in GeneiousPro 5.6 [85]. NJ and ML trees were constructed from these alignments using the same software.

### 4.2. Plant Materials and Growth Conditions

Wild-type *A. thaliana* ecotype Columbia (Col-0) and *A. chinensis* (‘Hort16A’) were obtained from existing seed stocks at PFR. Arabidopsis T-DNA insertion knockout lines (*atcpk3-1*, SALK_106720C; *atcpk3-2*, SALK_022862) and SAIL overexpressor lines of *AtCPK3* (SAIL-120-H09) were obtained as seed stocks from the Nottingham Arabidopsis Stock Centre (NASC, UK). Wild-type *O. sativa* L. ‘Nipponbare’ seeds were obtained from the National Institute of Agrobiological Sciences (NIAS) in Japan.

*A. thaliana* plants that overexpress *AtCPK3* were also developed using Gateway^®^ cloning technology into the binary vector pHEX2 under cauliflower mosaic virus (CaMV) 35S promoter control. Arabidopsis (*col-0*) plants were transformed with the pHEX2AtCPK3 constructs in *Agrobacterium tumefaciens* GV3101 by floral dipping. Cycles of seed collection and selection in MS Agar supplemented with 100 mg/mL kanamycin were carried out until the fourth (T4) generation to ensure homozygosity.

*A. chinensis* (‘Hort16A’) plants that are knockouts or overexpressors of *AcCPK16* were developed using *A. tumefaciens* strain EHA105 harboring binary plasmids pSAK778S_304838 (containing CaMV 35S promoter for overexpression) and pTKO2S_304838 (using intron insertion for gene knockout). Leaf strips (2–5 mm) excised from young leaves of tissue culture-grown shoots were inoculated with suspension cultures of *A. tumefaciens* containing either of the binary plasmid constructs. Regeneration and selection were carried out using a M1 medium containing 150 mg/L of kanamycin and 300 mg/L of timentin (GlaxoSmithKline, Melbourne, VIC, Australia.). Rooted plants from calli buds were transferred into soil and progressively acclimatized for three weeks before stress treatments.

Information about the insertion location among the obtained T-DNA lines from NASC has been reported by Mehlmer et al. (2010) [59]. The *atcpk3-1* seeds were homozygotes, while the *atcpk3-2* and SAIL *AtCPK3* seeds were T2/T3 segregating lines. To confirm the differences between wild-type, overexpressor, and knockout lines in Arabidopsis and kiwifruit, RT-PCR amplifying the full mRNA sequence was carried out, comparing *AtCPK3* or *AcCPK16* gene expression semi-quantitatively (Supplementary Figure S4).

All plants were grown in a Physical Containment Level 2 (PC2) containment glasshouse or growth cabinet at 22–26 °C with a 16 hr light and 8 hr dark cycle.

Transgenic *O. sativa* lines for *OsCPK1* and *OsCPK15* overexpression and gene knockout could not be generated at the time of experimentation due to limitations in containment permissions as well as the generation time required. There were also no genetic mutant stocks available.

### 4.3. Stress and Pathogen Treatments in A. thaliana

Arabidopsis seedlings were grown in soil with normal watering for three weeks. Drought treatment was carried out by water elimination for up to 21 days. Leaf tissue samples were collected from treated plants and controls at 7, 14, and 21 days. Salt stress treatment was carried out by germinating seeds in MS agar supplemented with 0 mM, 75 mM, and 150 mM NaCl. There were three biological replicates for each treatment and time point/salt concentration. For the phenotype analysis comparing wild-type and transgenic lines, there were ten biological replicates for each plant line.

For the *B. cinerea* treatment, two-week-old cultures (isolate REB 702-1) grown on potato dextrose agar (PDA) plates were flooded with 30 mM K_2_HPO_4_, 0.05% glucose to collect spores and incubated at 20–22 °C for 3 h. Four-week-old Arabidopsis plants were inoculated by placing a 5 µL drop of the spore suspension (1 × 10^5^ spores/mL) onto each of three rosette leaves. Leaf samples were taken from inoculated and mock-inoculated plants at 0, 1, 2, 6, and 10 dpi, with three biological replicates per time point. Infections were identified by the presence of lesions and sporulation on the leaves. For the phenotype analysis comparing wild-type and transgenic lines, fungal infection was applied as described, with ten biological replicates for each plant line.

For the Pto DC3000 treatment, stab culture stocks of *P. syringae* van Hall 1902 (ICMP18429, MPI Import Permit No. 2010039160) were obtained from the International Collection of Microorganisms from Plants (ICMP, Landcare Research New Zealand). Liquid culture was grown from this stock using Luria-Bertani (LB) broth at 28 °C for 48 hrs. Four-week-old Arabidopsis were inoculated by placing a 5 µL drop of the bacterial suspension (1 × 10^8^ CFU/mL) onto each of three rosette leaves. Mock inoculation was also performed using LB broth. Leaf samples were taken from inoculated and mock-inoculated plants at 1, 2, 6, and 10 dpi. Infections were identified by the presence of lesions on the leaves.

Infection of Arabidopsis plants with TMV and TYMV was carried out on three-week-old Arabidopsis seedlings containing six to eight rosette leaves. Virus-infected leaf tissue was homogenized in a phosphate buffer with carborundum (600 grit, BDH) and then rubbed gently onto three leaves in each plant. Mock inoculations were also performed with the inoculation buffer. Leaf samples were taken at 2, 3, 7, 14, 21, 28, and 35 dpi. Virus infection was confirmed by RT-PCR using virus-specific primers. For the phenotype analysis comparing wild-type and transgenic lines, only infection with TYMV was carried out, with ten plants inoculated per line.

### 4.4. Stress and Pathogen Treatments in O. sativa

Rice seedlings were grown in soil with normal watering until three weeks. Drought treatment was performed by eliminating watering in the succeeding 14 days. Leaf tissue samples were collected from treated plants and controls at 7 and 14 days, with three biological replicates for each treatment and time point.

For *M. grisea* and *P. syringae* pv*. syringae* (Pss) treatments and detached leaf assays were performed instead of whole plant infections due to facility restrictions. *M. grisea* (ICMP14481, MPI Import Permit No. 2001012667) and Pss (ICMP4265) stock cultures were obtained from ICMP (Landcare Research, NZ). *M. grisea* was subcultured on PDA plates, double bagged in ziplock bags and grown at 24–26 °C in the dark for 4 days and then under 12 h light/dark cycle for 7 days. Pss was subcultured in Kings medium B agar plates and grown at 24–26 °C for 48 hrs.

Spot inoculation of detached rice leaves was performed with modifications from a previous study [86]. To prepare the inoculum for *M. grisea*, established plates were flooded with 0.25% gelatine 0.02% Tween^®^ 20 solution and were filtered using sterile cheesecloth. The spore suspension was adjusted to 1 × 10^4^ spores/mL. An overnight liquid culture was prepared from established cultures using LB broth to prepare the inoculum for Pss. The bacterial suspension was adjusted to 1 × 10^5^ CFU/mL. The youngest leaves from each rice plant were selected and cut into 5 cm segments. The detached leaf segments were immediately placed into Petri dishes lined with moist filter paper. Each leaf segment was spot-inoculated with seven 5 µL droplets of either control, mock, conidial, or bacterial suspension. The Petri dishes were sealed, placed in a ziplock bag and maintained at 21 to 24 °C under continuous fluorescent light (10 to 12 μEm^−2^ s^−1^). Sterile deionized water was added every day to the filter paper to maintain moisture levels and avoid desiccation. Samples were taken at 2, 6, and 10 dpi, with three biological replicates per time point. Infections were identified by the presence of lesions.

Fresh CymMV-infected vanilla leaf tissue was used to treat the virus infection in rice. Inoculation was carried out as described in Arabidopsis. Leaf samples were taken at 2, 7, 14, 21, and 28 dpi, with three biological replicates per time point. Agdia Immunostrips^®^ monoclonal antibody strip systems (Agdia Inc., Elkhart, IN, USA) were used to detect CymMV, following the manufacturer’s instructions.

### 4.5. Stress and Pathogen Treatments in A. chinensis

Drought treatment was carried out for wild-type *A. chinensis* ‘Hort16A’ grown in soil, with three biological replicates for each treatment and time point. Before planting, kiwifruit seeds were stratified by soaking overnight in 10 ppm giberellic acid (GA3) to increase the germination rate. Kiwifruit seedlings were grown with normal watering until four weeks. Drought treatment was carried out by non-watering. Leaf tissue samples were collected at 7 and 14 days. For the phenotype analysis comparing wild-type and transgenic lines, drought treatment was carried out with only two biological replicates for each plant line due to the limited availability of viable transgenic plants.

For the fungal pathogen *B. cinerea,* treatment in kiwifruit inoculum preparation and inoculation was performed as described above. For the phenotype analysis comparing wild-type and transgenic lines, spot inoculation on detached leaves was carried out, with three biological replicates for each line. Measurements of fungal growth or leaf lesions were taken at 2 and 7 dpi.

For virus infection of kiwifruit, freeze-dried leaf tissue from a CMV-infected Delphinium plant (isolate 03/76) was inoculated into two leaves of each of four-week-old kiwifruit seedlings, as described above for Arabidopsis. Leaf samples were taken from inoculated and mock-inoculated plants at 7, 14, 21, 28, and 35 dpi. Agdia Immunostrips^®^ monoclonal antibody strip systems (Agdia Inc., Elkhart, IN, USA) were used to detect CMV, following the manufacturer’s instructions.

Inoculation of kiwifruit with *Pseudomonas* spp. could not be performed due to biological safety restrictions and experimental limitations in the facility.

### 4.6. RNA Extraction, Quality Analysis and cDNA Synthesis

Total RNA was extracted from 100 mg of liquid nitrogen-powdered leaf samples using a Spectrum TM Plant Total RNA Kit (Sigma-Aldrich, St. Louis, MO, USA) for Arabidopsis and rice and using a modified cetyltrimethylammonium bromide (CTAB) extraction procedure [87] for kiwifruit. RNA samples were treated with DNase I (amplification grade; Invitrogen, San Diego, CA, USA) to remove any potential genomic DNA (gDNA) contamination. RNA concentration and purity were measured using a Nanodrop ND-1000 spectrophotometer (Nanodrop Technologies Inc., Wilmington, DE, USA), while RNA integrity was analyzed using a Bioanalyzer 2100 RNA Nano LabChip 6000 (Agilent Technologies, Santa Clara, CA, USA). All RNA samples used for the succeeding experiments were ensured to have an absorbance ratio (A260/280) between 1.8 and 2.2 and an adjusted RIN value of 7.0 or greater. RNA samples were reverse transcribed into cDNA using a SuperScript^®^ VILO™ cDNA synthesis kit (Life Technologies-Invitrogen, San Diego, CA, USA) following the manufacturer’s protocol. The total amount of RNA transcribed to cDNA was adjusted to 2 µg in Arabidopsis and 1 µg in rice and kiwifruit, in a total volume of 40 µL.

### 4.7. Reference Gene Selection and RT-qPCR Primers

Reference genes were used as internal controls to ensure cDNA quality and as standards for quantifying stress and other stimulus-responsive genes. For Arabidopsis, the reference genes that were selected for evaluation in this study were: Elongation factor-1 α (*EF-1α*, At5g60390), SAND family protein (*SAND*, At2g28390), Protodermal factor 2 (*PDF2*, At1g13320) and F-Box family protein (*F-BOX*, At5g15710). For rice, the reference genes that were selected for evaluation were TBC1 domain family member 22A (*OsTBC*, LOC_Os09g34040), Tumor protein homolog (*OsTPH*, LOC_Os11g43900.1), RNA-binding protein (*OsRBP*, LOC_Os03g46770.1) and Expressed protein 1 (*OsEP1*, LOC_Os07g02340.1. For kiwifruit, the reference genes selected for evaluation were: Actin mRNA 1 (*AdACT1*, orthologue of At5g09810), Ubiquitin-conjugating enzyme 9 (*UBC9*, orthologue of At4g27960) and Protein Phosphatase 2A regulatory unit (*PPPRSA*, orthologue of At1g13320). All reference genes were analyzed using GeNORM [88].

Gene-specific primers near the 3′ end were designed for each of the targeted Group IIb CPK genes in Arabidopsis, rice, and kiwifruit. Design of forward and reverse primers was performed using the software Primer3Plus (version 3.3) [89] with the following criteria to ensure primer specificity and efficiency: (1) melting temperature (Tm) of 60 ± 3 °C; (2) primer length of 20 to 27 base pairs (bp); (3) GC content of 45–55%, and (4) amplicon size of 130–200 bp. The target regions of the forward and reverse primers spanned an intron (to detect genomic DNA contamination). Primers for the reference genes for Arabidopsis were adapted from previous reports mentioned above [90]. A list of primers used for RT-qPCR is provided in Supplementary Table S1.

### 4.8. RT-qPCR Analysis

To quantify the transcript accumulation of the target and reference genes from each sample, qPCR reactions were performed using a LightCycler 480 Real-Time PCR system (Roche Applied Science, Branchburg, NJ, USA). Reactions were in a 10 μL total volume containing 1 μL of primer pair (2 μM forward and reverse primer), 4 μL of cDNA and 5 μL of LightCycler 480 SYBR Green I Master mix reagent. A Biomek 3000 Robot (Beckman Coulter, Fullerton, CA, USA) was used to aliquot all reagents, primers, and samples into 384-well plates, with two technical replicates and three biological replicates for each sampling time point. The qPCR reaction consisted of pre-incubation at 95 °C for 5 min and amplification with 45 cycles of denaturing at 95 °C for 10 s, annealing at 60 °C for 10 s and extension at 72 °C for 10 s. Fluorescence acquisition was set up at the end of each cycle. The amplification step was followed by a melting curve analysis, with one cycle of 95 °C for 5 s, 65 °C for 1 min and a ramp to 97 °C at a rate of 0.11 °C/s. Five fluorescence acquisitions per °C were taken. Samples were cooled at 40° for 10 s.

Fluorescence data per cycle were exported from the LightCycler 480 software into a *.csv file using Python 2.6.3 (Python Software Foundation; custom script by Jeremy McRae, PFR). Baseline correction, log transformation and primer PCR efficiency calculation from linear regression were done using the software LinRegPCR 11.1. The M value for each reference gene and the Normalization Factor for each Q value were calculated using GeNorm v3.5 analysis software. Reference genes with an M value less than 1 were considered acceptable for use in the normalization of qPCR data. Transcript accumulation of the targeted CPK genes was normalized using two to three reference genes (three for Arabidopsis and kiwifruit, two for rice).

### 4.9. Statistical Analysis

Statistical support for the results was determined using Analysis of Variance (ANOVA) and follow-up tests such as Tukey’s test and Fisher’s LSD. Statistical support was considered strong (*p* ≤ 0.01), good (0.01 < *p* ≤ 0.05) or weak (0.05 < *p* < ~0.10). Levene’s test was initially done before the ANOVA test to determine if the values have equal variance. The statistical software Minitab was used to perform all statistical tests (Minitab 17 Statistical Software 2010).

## Data Availability

Data is contained within this article. Raw data are available on request.

## Supplementary Materials

The following supporting information can be downloaded at: https://www.mdpi.com/article/10.3390/plants14020294/s1, Figure S1: Seed germination among wild-type Arabidopsis and *AtCPK3* transgenic plants in different salt concentrations.; Figure S2: Seed germination among wild-type Arabidopsis and *AtCPK3* transgenic plants in different salt concentrations; photos of representative plates.; Figure S3: Phenotype analysis of the wild type and transgenic lines of Arabidopsis and kiwifruit under fungal infection (*Botrytis cinerea*).; Figure S4. RT-PCR results comparing gene expression between wild-type, overexpressor, and knockout Arabidopsis plants. (a) Arabidopsis transgenic lines: Lanes 1 to 3: Overexpressors developed from pHEX2AtCPK3.3; Lane 4: Wt Arabidopsis; Lane 5 to 7: *AtCPK3* knockouts SALK_106720C (*atcpk-*1), SALK_022862 (*atcpk-2*), and SALK_095134 (atcpk-3); Lane 8: *AtCPK3* overexpressor SAIL-120-H09. Lane 9: NTC; Lane 10: 100 bp ladder (Solis BioDyne) (b) Kiwifruit transgenic lines: Lane 1: 1 Kb plus DNA ladder (Invitrogen); Lane 2: WT kiwifruit; Lane 3: *AcCPK16* OX E05; Lane 4: *AcCPK16* OX E06; Lane 5: *AcCPK16* KO E05; Lane 6: *AcCPK16* KO E10; Lane 7: *AcCPK16* KO E11; Lane 8: NTC.; Supplementary Table S1. Primers used for RT-qPCR analysis of target and reference genes.
