# Supplementary figures and images for: Arabidopsis Calcium Dependent Protein Kinase 3, and Its Orthologues OsCPK1, OsCPK15, and AcCPK16, Are Involved in Biotic and Abiotic Stresses
Source: Plants (Basel). 2025 Jan 20;14(2):294. doi: 10.3390/plants14020294 (PMC11768100; doi:10.3390/plants14020294)

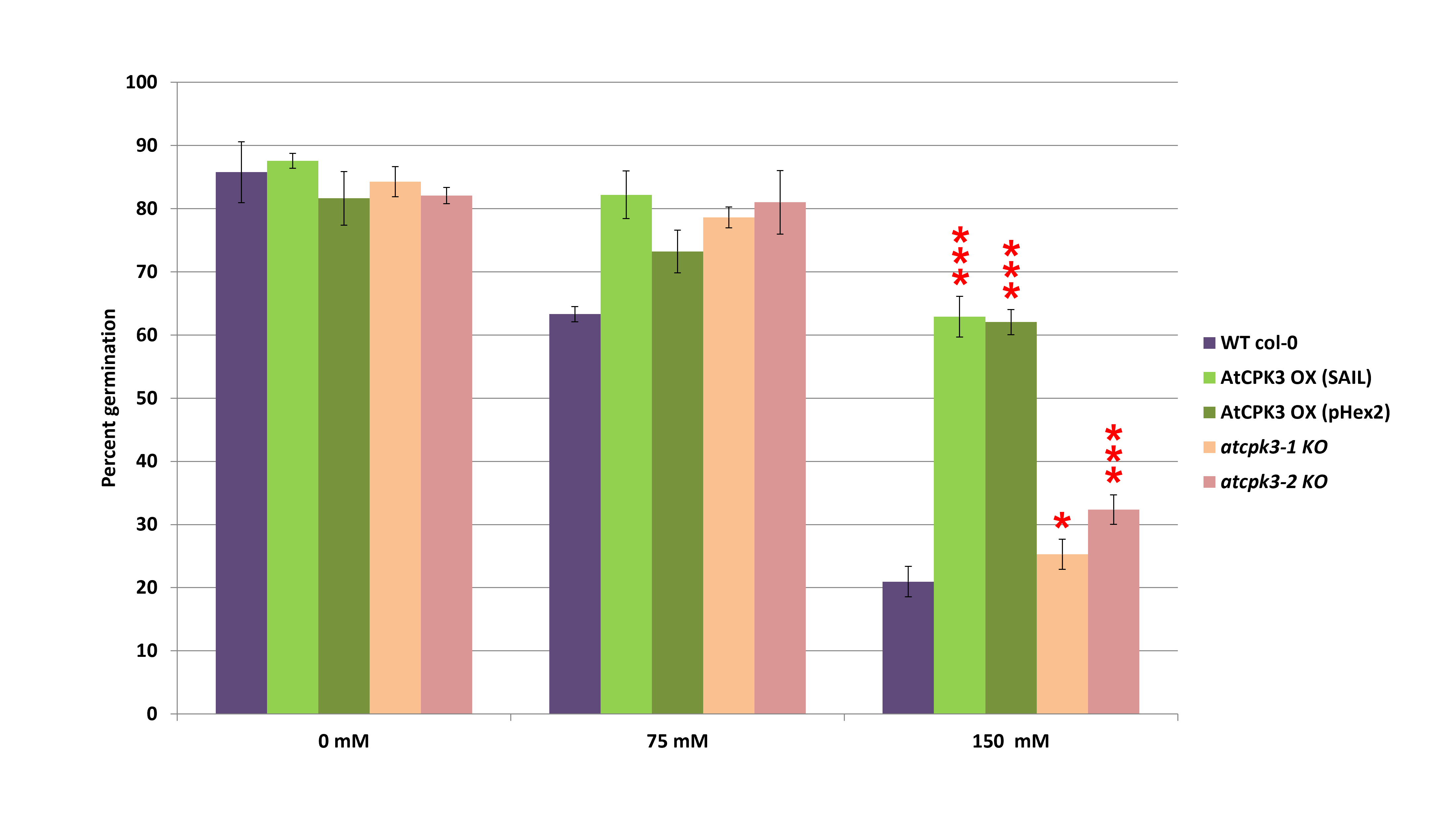

Supplement: Supplementary file 1 [file plants-14-00294-s001.zip › Figure S1.tif]

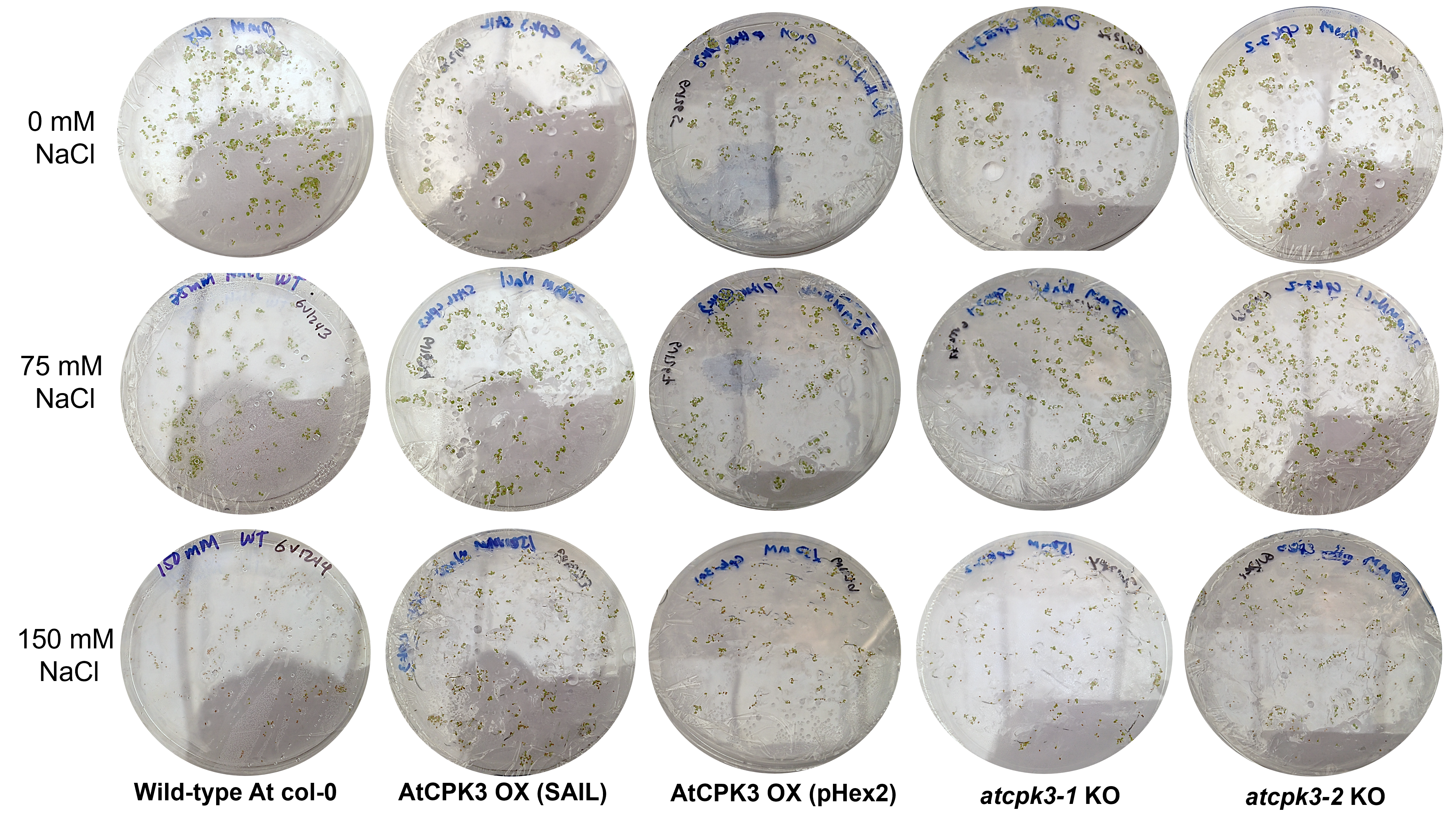

Supplement: Supplementary file 1 [file plants-14-00294-s001.zip › Figure S2.tif]

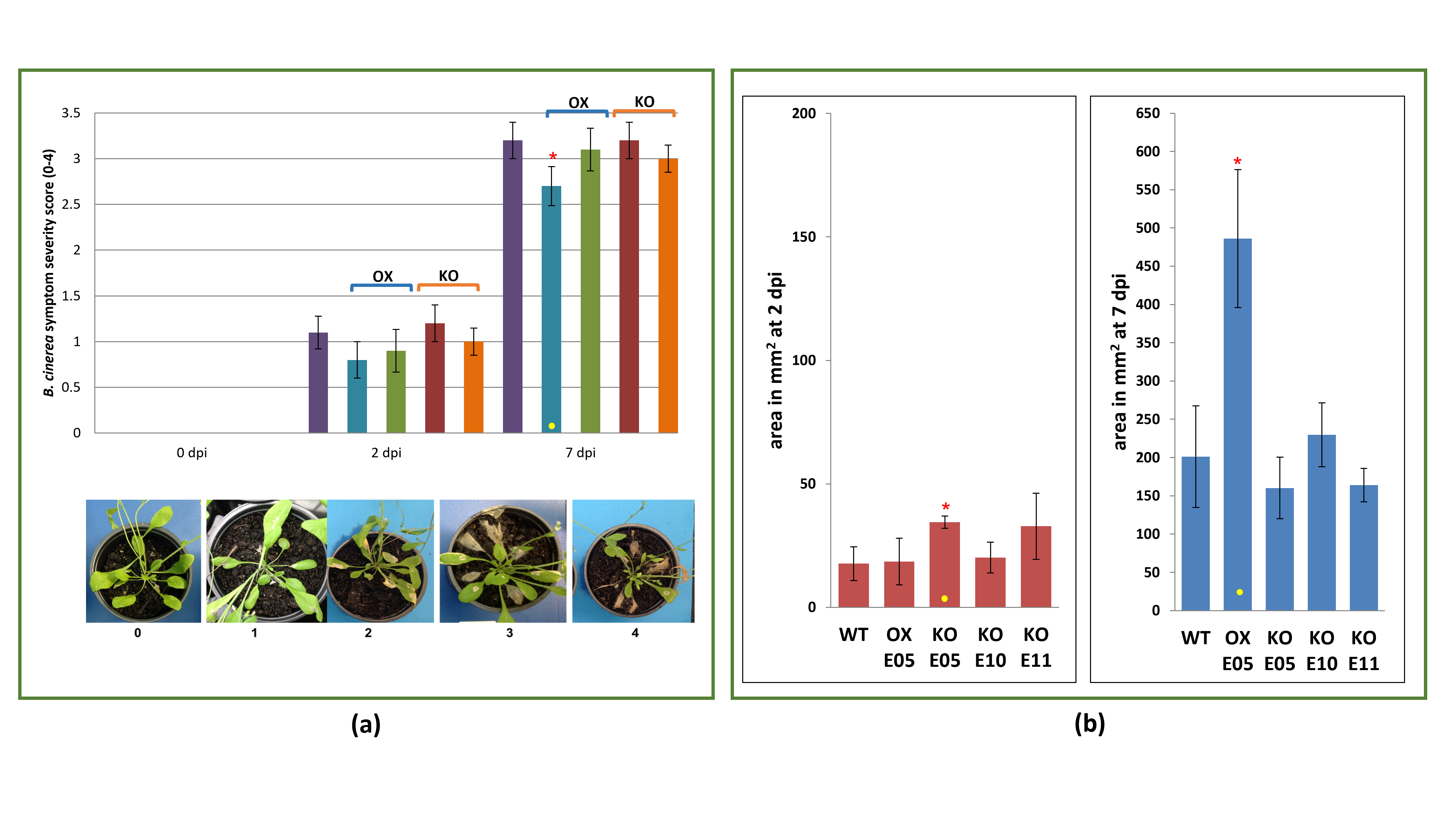

Supplement: Supplementary file 1 [file plants-14-00294-s001.zip › Figure S3.tif]

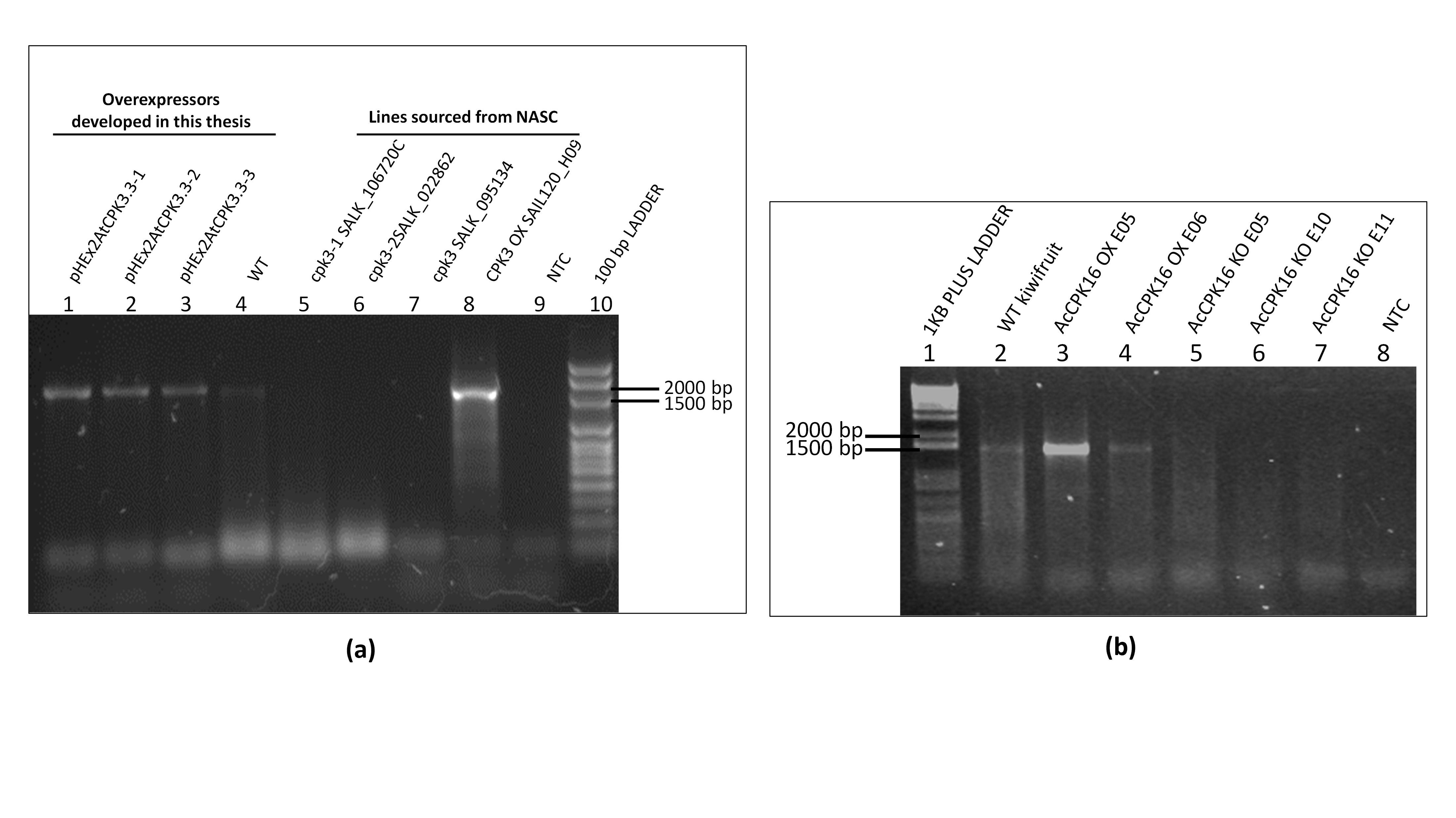

Supplement: Supplementary file 1 [file plants-14-00294-s001.zip › Figure S4.tif]

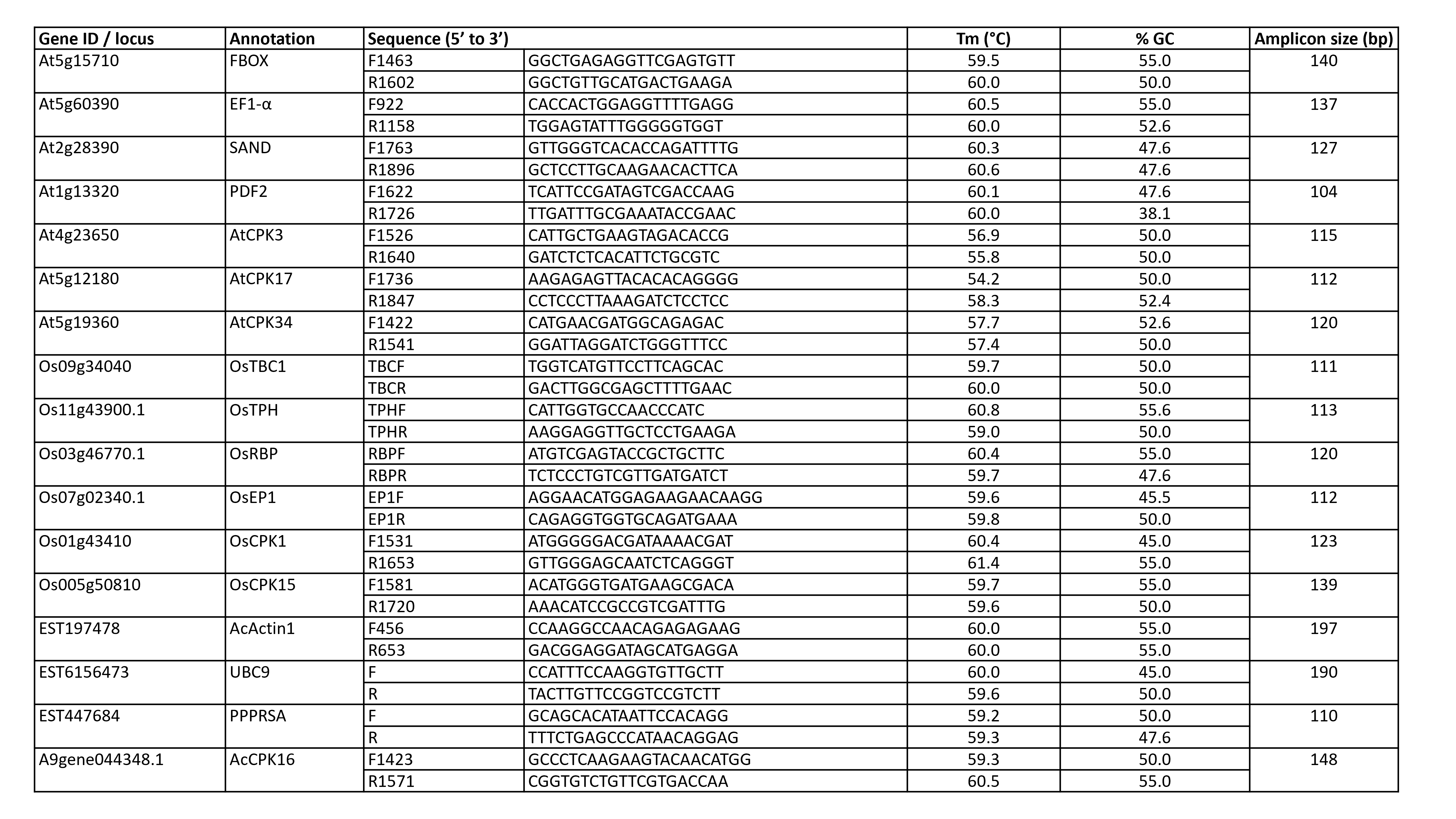

Supplement: Supplementary file 1 [file plants-14-00294-s001.zip › Table S1.tif]
